# Electronic-cigarette use among young people in Wales: evidence from two cross-sectional surveys

**DOI:** 10.1136/bmjopen-2014-007072

**Published:** 2015-04-01

**Authors:** Graham Moore, Gillian Hewitt, John Evans, Hannah J Littlecott, Jo Holliday, Nilufar Ahmed, Laurence Moore, Simon Murphy, Adam Fletcher

**Affiliations:** 1 Centre for the Development and Evaluation of Complex Interventions for Public Health Improvement (DECIPHer), School of Social Sciences, Cardiff University, Cardiff, UK; 2 South East Wales Trials Unit (SEWTU), School of Medicine, Cardiff University, Cardiff, UK; 3 MRC/CSO Social & Public Health Sciences Unit, University of Glasgow, Glasgow, UK

**Keywords:** PUBLIC HEALTH, PREVENTIVE MEDICINE, Smoking

## Abstract

**Objectives:**

To examine the prevalence of electronic(e)-cigarette use, prevalence of e-cigarette and tobacco use by age, and associations of e-cigarette use with sociodemographic characteristics, tobacco and cannabis use among young people in Wales.

**Design:**

Data from two nationally-representative cross-sectional surveys undertaken in 2013–2014. Logistic regression analyses, adjusting for school-level clustering, examined sociodemographic characteristics of e-cigarette use, and associations between e-cigarette use and smoking.

**Setting:**

Primary and secondary schools in Wales.

**Participants:**

Primary-school children aged 10–11 (n=1601) and secondary-school students aged 11–16 (n=9055).

**Results:**

Primary-school children were more likely to have used e-cigarettes (5.8%) than tobacco (1.6%). Ever use of e-cigarettes remained more prevalent than ever use of tobacco until age 14–15. Overall, 12.3% of secondary-school students (aged 11–16) reported ever using e-cigarettes, with no differences according to gender, ethnicity or family affluence. The percentage of ‘never smokers’ reporting having used e-cigarettes was 5.3% at age 10–11 to 8.0% at age 15–16. The proportion of children who had ever used an e-cigarette and reported currently smoking increased from 6.9% among 10–11 year olds to 39.2% in 15–16 year olds. Only 1.5% (n=125) of 11–16 year-olds, including 0.3% of never smokers, reported regular e-cigarette use (use at least once a month). Current weekly smokers were 100 times more likely than non-smokers to report regular e-cigarette use (relative risk ratio (RRR=121.15; 95% CI 57.56 to 254.97). Regular e-cigarette use was also more likely among those who had smoked cannabis (RRR 53.03; 95% CI 38.87 to 80.65).

**Conclusions:**

Many young people (including never-smokers) have tried e-cigarettes. However, regular use is less common, and is associated with tobacco cigarette use. Longitudinal research is needed to understand age-related trajectories of e-cigarette use and to understand the temporal nature of relationships between e-cigarette and tobacco use.

**Strengths and limitations of this study**

- Data derived from two large, nationally-representatives data sets provide evidence that many young people, including non-smokers aged 10–16 in Wales have used e-cigarettes (5.8% of 10–11 year olds; 12.3% of 11–16 year olds).
- Ever use of e-cigarettes is more common than ever use of tobacco for all groups except the oldest secondary-school age group (age 15–16 years).
- Only 1.5% of young people aged 11–16 report regular e-cigarette use (ie, use of an e-cigarette at least monthly), which was strongly associated with smoking tobacco and cannabis.
- More detailed measures of e-cigarette use, and longitudinal data to understand temporal relationships between e-cigarette and tobacco use, are priorities for further research in this area.

## Background

Electronic cigarettes, or ‘e-cigarettes’, are hand-held devices that deliver smokeless nicotine through a battery-powered vaporisation process. Although not regulated or licensed as smoking cessation devices, e-cigarettes are seen by some as offering significant harm reduction potential where used effectively by adult smokers to support smoking cessation.^1–3^ While research on their role in smoking cessation is underdeveloped, there is some emerging evidence that e-cigarettes do offer promise for this purpose.^4^ ^5^ Hence, due to their perceived harm reduction potentials, many public health experts are now urging the WHO not to support their regulation as tobacco products or restrictions on their marketing.^6^

However, there are also significant concerns that, if unregulated, and marketed to young people, e-cigarettes could seriously undermine the success of recent tobacco control strategies. The carcinogens and other toxins within e-cigarettes are one concern;^1^ harm reduction arguments do not hold where e-cigarettes are used by young people who would not otherwise have been using tobacco. Furthermore, it has been suggested that e-cigarette use could act as a new gateway into nicotine addiction and tobacco use for young people.^7^ ^8^ While insufficient time has passed since the proliferation of e-cigarettes for longitudinal studies to emerge, cross-sectional studies have indicated associations between e-cigarette use and intentions to smoke tobacco.^7^ ^9^

There is emerging international evidence that e-cigarettes are being used by growing numbers of young non-smokers.^7^ ^8^ ^10–13^ For example, the 2011 and 2012 USA Youth Tobacco Surveys revealed that ever use of e-cigarettes among 11–18 year-olds more than doubled in 1 year, from 3.3% in 2011, to 6.8% in 2012.^7^ Further analyses found that e-cigarette use among this population did not appear to support cessation and raised concerns that it was more likely to have adverse health effects via subsequent use of conventional cigarettes.^12^ Another recent US study of more than 1500 high school students found that e-cigarette users were more likely to report using cannabis ‘blunts’.^14^ In France, e-cigarette use is now reported by 6% of 12–14 year-olds, 12% of 15–16-year-olds, and 9% of 17-year-olds.^8^ In this study the majority (64.4%) of 12–14 year-old e-cigarette users were non-smokers.^8^

Tobacco companies have increasingly invested in the e-cigarette market and some have expressed concerns regarding a perceived rapid increase in aggressive, youth-focused marketing of e-cigarettes.^2^ ^15^ The latest generation of e-cigarette products offer availability in a wide range of colours and flavours.^16^ These colours and flavours may appeal to adults.^17^ However, it is likely that these will appeal particularly to young people, as has been the case with sweet-flavoured alcoholic drinks, which have been targeted largely toward young people.^18^ Recent advertising campaigns and product placements closely resemble the messages used by the tobacco industry to target young people in the 1950s and 1960s.^19^ There is also concern that the visibility of e-cigarettes in places where the marketing or use of tobacco has been banned may reverse efforts to denormalise smoking as a strategy to reduce the uptake of smoking.^20^

Hence, some have voiced concerns that, while presenting itself as a partner in harm reduction, the tobacco industry is seizing new opportunities to promote nicotine addiction among young people and recruit potential smokers.^21^ ^22^ Some public health authorities in the USA have responded to concerns regarding the growing visibility of e-cigarettes by banning their use in public places.^23^ In the UK, the Welsh and Scottish Governments have recently issued consultation documents discussing potential legislation.^21^ ^24^ The Department of Education's Children and Families Bill also now contains provisions to prevent the sale of e-cigarettes to anyone aged under 18.^25^ However, due to the longevity of their life-span and availability online, prohibition of e-cigarettes will be very hard to enforce and is extremely unlikely to be an effective prevention measure in isolation. To develop comprehensive prevention strategies, we therefore urgently need to better understand youth e-cigarette use, including how this relates to smoking.

Despite increasing policy and public interest, young people's use of e-cigarettes in the UK remains a major empirical blindspot. There is some emerging evidence of youth e-cigarette use in the UK, including among non-smokers.^9^ ^26^ ^27^ For example, in Wales in 2013–2014, an online (and non-representative) survey of 740 young people (aged 13–18) found that more than one-fifth of young people reported having used an e-cigarette at least once and 5% reported using them weekly or more, although regular use among non-smokers was approximately 1%.^27^ However, there have been no large-scale, nationally representative surveys of young people's e-cigarette use, which are required to estimate prevalence, average age of initiation and examine the characteristics of young people who have used an e-cigarette. Furthermore, no studies outside of the USA have examined associations with youth cannabis smoking.

Drawing on data from two large-scale surveys of young people undertaken in Wales in 2013–2014, this paper aims to estimate the prevalence of e-cigarette use among young people in Wales, and to examine associations with sociodemographics and tobacco and cannabis use. One of these surveys recruited a large nationally representative sample of primary school children aged 10–11 and therefore can assess the level of initiation of e-cigarette use at a young age. The second survey involves a large nationally representative sample of secondary school-children. Hence, in combination, these data can provide the most robust prevalence estimate of youth e-cigarette use in a UK country to date, facilitating comparison of age-related trajectories of tobacco and e-cigarette use, and associations between e-cigarette use and smoking (tobacco and cannabis) in order to inform further research and more appropriate policy responses in this area.

## Methods

This study combines two data sets: the CHild Exposure to Tobacco Smoke (CHETS) survey undertaken in Wales (‘CHETS Wales 2’) in 2014; and the 2014 Welsh Health Behaviour in School-aged Children (HBSC) Survey (‘HBSC Wales’).

### CHETS Wales 2: study design and recruitment

CHETS Wales 2 was a cross-sectional study of Welsh school children in year 6 (age 10–11) within a nationally representative sample of 75 primary schools. It replicated earlier surveys conducted in Wales in 2007 and 2008, which examined child secondhand smoke exposure before and after introduction of Welsh smoke-free legislation (‘CHETS Wales’),^28^ and was commissioned and powered primarily to investigate changes in child exposure to smoke in cars since 2008. For CHETS Wales, state maintained schools with year-6 students were stratified according to high/low free school meal entitlement (above and below the national median of 17.12% of children within the school entitled to a free lunch on the basis of a low family income) and local education authority. Within each stratum, schools were selected on a probability proportional to school size. These same 75 schools that participated in CHETS Wales were contacted by letter and invited to take part in CHETS Wales 2; where schools declined, replacement schools were identified from the same stratum. Schools received £50 for participating in CHETS Wales 2. If there was more than 1 year-6 (final year primary school) class within the school 1 year-6 class was randomly selected by the research team to participate. Data were collected between February and April 2014. Two researchers attended each data collection to ensure sufficient support and assistance where required. Teachers were asked to be present, but to remain at the front of the classroom and not to intervene in the data collection in any other way.

### HBSC Wales: study design and recruitment

HBSC Wales was a cross-sectional study of Welsh school students aged 11–16 in a nationally representative sample of 82 secondary schools. Wales is one of a number of countries participating in the HBSC study internationally.^29^ ^30^ It replicated previous HBSC surveys conducted in Wales,^31^ and was commissioned and powered primarily to track national and international changes in health behaviours among school-aged students. All maintained and independent secondary schools in Wales were stratified by local authority and eligibility for free school meals. Schools were selected using probability proportionate to size and that there was an element of disproportionate stratification to allow analysis at local health board (LHB) level. School head teachers were invited to take part in the HBSC survey by letter and followed up with telephone calls. Participating schools received £150 to cover any costs incurred due to participating. Within each participating school, one mixed ability class (approximately 25 students) from each school year 7–11 was randomly selected by the school to participate. Data were collected between November 2013 and February 2014. Trained fieldworkers attended each data collection to ensure sufficient support and assistance where required. Teachers were present during data collection but remained at the front of the classroom so they cannot see students’ responses.

### Measures

#### Sociodemographics

In both studies participants indicated their sex and year and month of birth. To measure socioeconomic status, young people completed the Family Affluence Scale (FAS)^32^ comprising measures of bedroom occupancy, car and computer ownership and family holidays which were summed to give an overall measure of family affluence. In the HBSC Wales survey young people were also asked which of the following best described them: White; Mixed Race; Asian or Asian British; Black or Black British; Chinese; or Other.

#### Use of e-cigarettes

In CHETS Wales 2, children were asked if they had ever used an e-cigarette, with response options of: ‘no’; ‘yes, once’; or ‘yes more than once’. In HBSC Wales, young people were asked if they had ever used an e-cigarette, with response options of: ‘I have never used or tried e-cigarettes’; ‘I have used e-cigarettes on a few occasions (1–5 times)’; or ‘I regularly use e-cigarettes (at least once a month)’. For analyses drawing on only the CHETS Wales 2 data or using both the data sets a binary variable (“ever used e-cigarettes”) was used. For CHETS Wales 2, this includes all respondents who reported ‘yes, once’ or ‘yes, more than once’. For HBSC Wales, that is respondents who either reported ‘I have used e-cigarettes on a few occasions (1–5 times)’ or ‘I regularly use e-cigarettes (at least once a month)’. As regular e-cigarette use was also measured in the HBSC Wales study, analyses using only the HBSC data examine a three category e-cigarette use variable (never, occasional and regular use).

#### Lifetime prevalence and frequency of current smoking

Lifetime smoking was measured in CHETS Wales 2 by asking children whether they ever smoked tobacco, with response options of ‘yes’ or ‘no’. In the HBSC Wales survey, respondents were asked ‘On how many days (if any) have you smoked cigarettes?’, with seven response options: ‘Never’; ‘1–2 days’; ‘3–5 days’; ‘6–9 days’; ‘10–19 days’; ‘20–29 days’; ‘30 days or more’. Respondents who report ‘never’ smoking cigarettes are compared to the other response options to assess lifetime prevalence. In both surveys, current smoking was assessed by asking ‘How often do you smoke tobacco at present?’, with response options of ‘every day’, ‘at least once a week, but not every day’, ‘less than once a week’, and ‘I do not smoke’.

#### Cannabis use

In the HBSC Wales survey, there was an item on drug use in which young people reported whether they had ever used cannabis or not.

### Research ethics

In both studies: schools signed and returned a commitment form to participate in the study; parents were sent information sheets and had the option of withdrawing their child from data collection (apart from at one school in CHETS Wales 2, which requested an ‘opt-in’ consent procedure whereby parents/carers informed their child's school if they *did* wish their child to participate in the study); and, at the start of each data collection session, participants were asked to provide active assent after having read an information sheet and having had the study explained. All young people had the opportunity to withdraw from the data collection session at any time.

### Statistical analysis

First, drawing on both data sets, the percentages of young people who have ‘ever used e-cigarettes’ within each school year are presented graphically, alongside the percentage who report having smoked tobacco, and the percentage of never smokers who report having used e-cigarettes. Second, the percentages of young people who report that they have used an e-cigarette and smoked tobacco (ever and current smokers) are also presented graphically by year group. To allow comparison of smoking by year group among young people who had used e-cigarettes with the sample as a whole, the percentage of smokers in each year group are also plotted in this second figure.

Binary logistic regression models are then used to examine associations of demographic variables and tobacco use with e-cigarette use among CHETS Wales 2 respondents. For HBSC Wales, multinomial logistic regression models are used to examine associations of demographic variables and tobacco use with e-cigarette use, with ‘occasional’ and ‘regular use’ compared against the reference category of ‘never used an e-cigarette’.

## Results

Overall, 114 schools were invited to participate in CHETS Wales 2 before the target sample of 75 schools was reached (response rate=65.8%). Of the 1862 pupils within selected classes, completed questionnaires were obtained from 1601 (86%). In schools where opt-out consent procedures were followed (n=74 schools, 1810 pupils), 56 children were withdrawn from the study by their parents, 35 children refused, and 141 were absent on the day of data collection, with data obtained from 1578 pupils (87.2%). In the one school which requested opt-in consent, this was provided for 23 of 52 children (44.2%), all of whom provided data.

Overall, 181 schools were invited to take part in HBSC Wales and 82 took part (response rate=45.3%). Of the remainder five were not eligible, 78 refused and 16 agreed to take part but convenient data collection times could not be identified. A total of 39 young people were withdrawn from the study by their parents via the system of opt-out consent, 33 refused to participate, and 772 were absent on the day of data collection, with data obtained from 9055 pupils (91.5%). Sociodemographics, e-cigarette use prevalence and the smoking characteristics of all participants are described in table 1.

**Table 1:** Sociodemographics, e-cigarette use and smoking characteristics of participants

	CHETS Wales 2 (N=1601)	HBSC Wales (N=9055)
Sociodemographic characteristics		
Mean (SD) age	10.9 (0.4)	13.3 (1.5)
Per cent (n) female	50.2 (803)	50.1 (4459)
Per cent (n) BME	NA	7.8 (630)
Mean(SD) FAS	6.0 (1.8)	6.8 (1.7)
E-cigarette use % (n)		
I have never used e-cigarettes	94.2 (1409)	87.7 (7932)
Ever used e-cigarettes	5.8 (87)	12.3 (1018)
Regular e-cigarette use	–	1.5 (125)
Smoked cigarettes (lifetime prevalence) % (n)		
Ever smoked cigarettes	1.6 (25)	12.1 (1017)
Frequency of tobacco use among current smokers % (n)		
Do not smoke	99.3 (1581)	94.7 (8564)
Less than once a week	0.4 (7)	1.8 (166)
At least once a week	0.2 (3)	1.1 (95)
Every day	0.1 (2)	2.5 (204)
Cannabis use (lifetime prevalence) % (n)		
Ever used cannabis	NA	7.4 (557)

BME, Black and Minority Ethnicity; CHETS, CHild Exposure to Tobacco Smoke; FAS, Family Affluence Scale; HBSC, Health Behaviour in School-aged Children; NA, not applicable.

Within the sample of 1601 children participating in the CHETS 2 survey, the mean (and SD) age was 10.9 (0.4) years. Items on e-cigarette use were completed by 1495 (93.4%) of children. There were no significant differences between children who did or did not complete questions on e-cigarette use, in terms of age, socioeconomic status or parental smoking. E-cigarette questions were completed by slightly fewer boys than girls (p<0.01), though overall, an approximately even gender balance was maintained (48.6% boys; 51.4% girls). The prevalence of ever using e-cigarettes among these year-6 primary school children was 5.8% (n=87), with the majority (3.7%; n=55) reporting that they only used them once and only 2.1% (n=32) of respondents reporting using them more than once. Fewer than 2% (n=25) of all 10–11 year olds reported having ever smoked, while less than 1% (n=12) reported current smoking.

Within the HBSC sample of 9055 young people, the mean age was 13.3 (SD 1.5) years. Although 98.9% (n=8950) of children completed e-cigarette questions, non-completion was slightly higher among boys and those who had smoked tobacco. The prevalence of e-cigarette use among secondary schools students was 12.3%, and 1.5% of these young people reported using them regularly (at least once a month). Overall, 12.1% of the secondary school students (n=1017) reported ever having smoked, although only 5.4% were current smokers. Lifetime prevalence of cannabis use was 7.4%.

**Figure 1:**
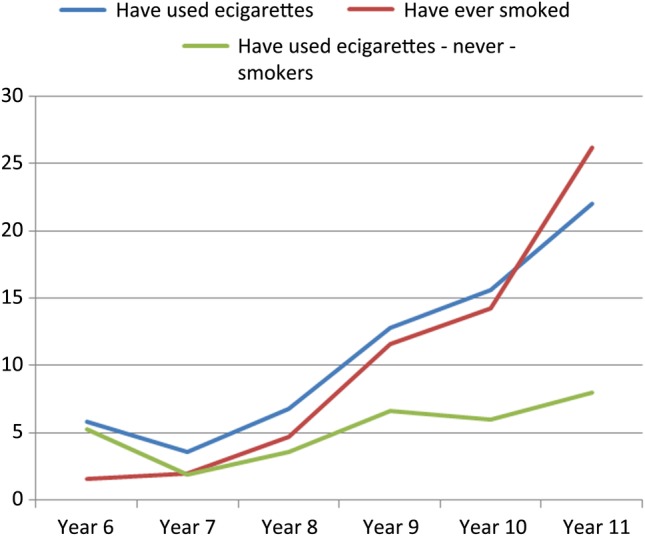
Percentage of young people reporting ever using e-cigarettes or smoking by school-year group.

Figure 1 shows the proportion of young people who report ever using e-cigarettes or smoking by school-year group, as well as tracking the use of e-cigarettes among ‘never smokers’. All these rates rise in parallel, with e-cigarette use slightly more common than tobacco use until school-year 11 (aged 15–16), when it is overtaken by smoking. The percentage of ‘never smokers’ who report having used an e-cigarette is 5% at age 10–11 (year 6), dropping to 2% in year 7, before rising throughout secondary school to 8% by age 15–16 (year 11).

**Figure 2:**
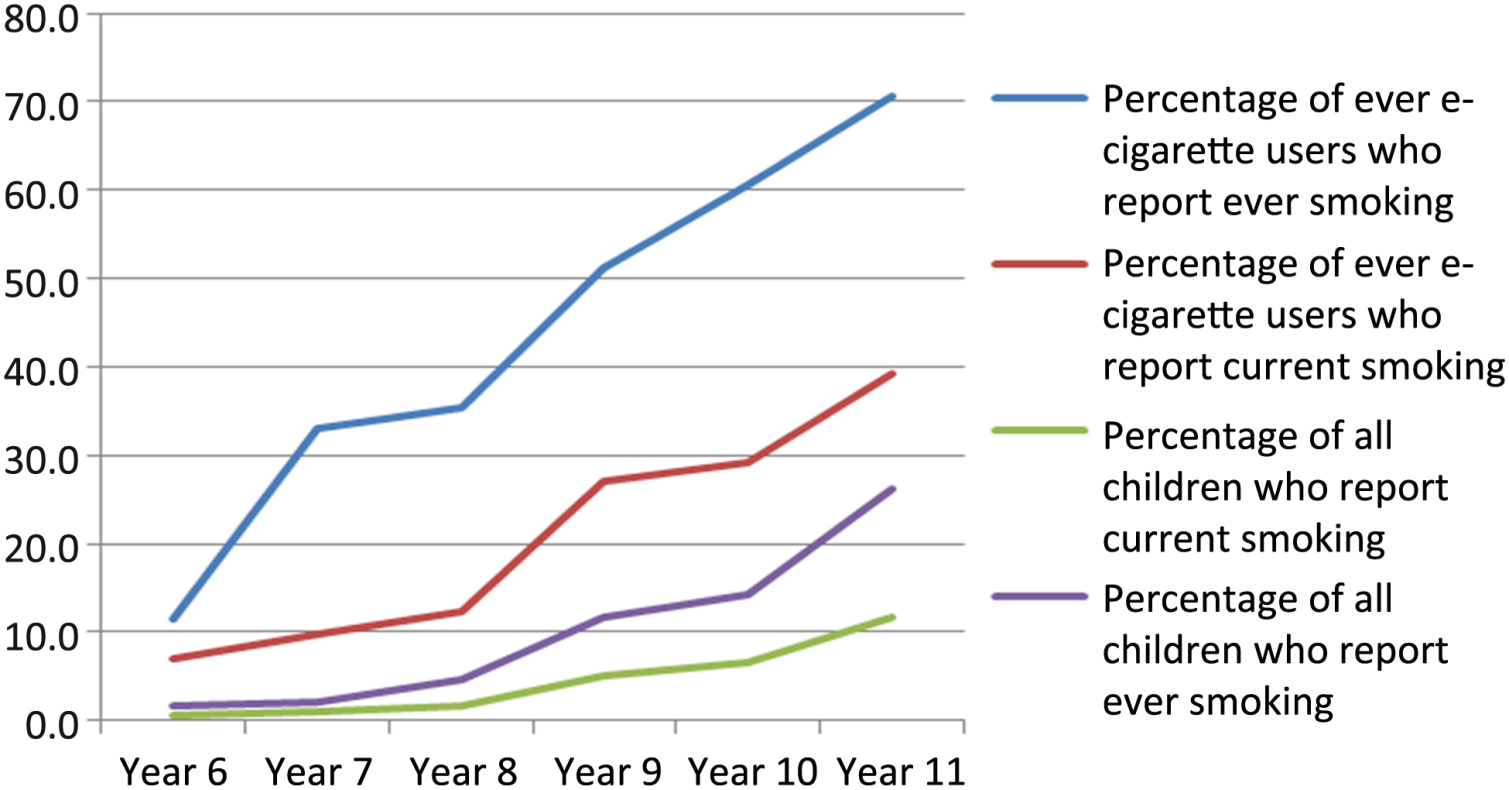
Percentage of young people reporting smoking (ever and current) among ever e-cigarette users and overall by school-year group.

Figure 2 shows the percentage of young people who had ever used an e-cigarette who report having used tobacco, by year-group (both ever smoking and current smoking), as well as overall prevalence of smoking across these years as a comparator. Through school-years 6, 7 and 8, the majority of children who had ever used an e-cigarette reported that they had never smoked tobacco. Among year 9s (age 13–14), about half of ever users of e-cigarettes have tried tobacco, whereas by school-years 10 and 11 a clear majority have tried tobacco. For all year groups, a minority of ever users of e-cigarettes report being current smokers, although this increases from 10% at age 10–11 (year 6) to 40% by age 15–16 (year 11). Figure 2 also shows the percentage of all respondents who report having tried smoking, or report being current smokers, indicating that at all ages, tobacco use is substantially higher among children who have used e-cigarettes than among those who have not. By age 16 for example, while a minority of all young people have tried smoking, more than two-thirds of those who have used e-cigarettes report that they have also tried smoking.

Among children aged 10–11 participating in CHETS Wales 2, e-cigarette use was more prevalent among boys (7.2% vs 4.6%; p =0.03) and there was a (non-significant) trend for greater use among children from less affluent families (table 2). The odds of e-cigarette use are more than 16 times greater for children aged 10–11 who had ever smoked tobacco, while among current smokers the odds of e-cigarette use were more than 17 times greater.

**Table 2:** Associations between e-cigarette use and sociodemographic and smoking characteristics among children aged 10–11 (CHETS Wales 2)

	Ever used e-cigarettes			
	% (n)	OR	95% CI	p Value
Gender				
Male	7.2 (52)	–	–	0.03
Female	4.6 (35)	0.62	0.40 to 0.96	
FAS				
NA	NA	0.91	0.79 to 1.06	0.22
Ever smoked cigarettes				
No	5.3 (77)	–	–	<0.01
Yes	47.6 (10)	16.41	8.24 to 32.70	
Current tobacco use				
Not a current smoker	5.5 (81)	–	–	<0.01
Current smoker				
<Weekly	57.1 (4)	17.23	6.16 to 48.19	
Weekly	66.7 (2)			
Every day	0			

FAS, Family Affluence Scale; NA, not applicable.

In the HBSC Wales sample of young people aged 11–16, e-cigarette use was almost as prevalent among young women as young men with no statistically significant differences according to gender. There were also no differences in e-cigarette use by ethnicity or, once again, according to family affluence (table 3). There was a very clear patterning according to lifetime smoking: almost half of those who had tried smoking have tried an e-cigarette (compared to only 4.8% of those who have never tried tobacco). Nevertheless, 42.8% of young people who had used e-cigarettes on a few occasions reported that they had never smoked tobacco. Regular e-cigarette use was more likely among those who had smoked tobacco, both in terms of relative risk ratio (RRR 66.30) and absolute values, with 80% of regular e-cigarette users reporting having also smoked tobacco. Current smoking was also strongly associated with e-cigarette use: RRRs for regular e-cigarette use among young people smoking weekly (RRR=121.15; 95% CI 57.56 to 254.97) or daily (RRR=115.38; 95% CI 70.09 to 189.91) both being over 100. However, 72.1% of young people who had used an e-cigarette a few times, and 43.2% of regular e-cigarette users, were from the larger group of young people who were not current smokers (hence while current smoking is associated with a greater relative risk of e-cigarette use, most young people who have used an e-cigarette are not smokers). As with lifetime smoking, lifetime cannabis use was strongly associated with experimental e-cigarette use (RRR 20.01; 95% CI 16.26 to 24.64) and regular e-cigarette use (RRR 53.03; 95% CI 34.87 to 80.65).

**Table 3:** Associations between frequency of e-cigarette use and sociodemographic and smoking characteristics (HBSC Wales)

	I have tried e-cigarettes on a few occasions (1–5 times)				Regular e-cigarettes (at least once a month)			
	Weighted % (n)	RRR	95% CI	p Value	Weighted % (n)	RRR	95% CI	p Value
Gender								
Male	11.2 (471)	–	–	–	1.8 (74)	–	–	–
Female	10.4 (417)	0.89	(0.71 to 1.11)	0.28	1.2 (50)	0.68	0.40 to 1.13	0.14
Ethnicity								
White	10.8 (824)	–	–	–	1.5 (114)	–	–	–
No-white	11.6 (67)	1.10	0.83 to 1.47	0.50	1.4 (10)	1.19	0.63 to 2.27	0.59
FAS								
	–	0.98	0.94 to 1.02	0.35	–	1.07	0.94 to 1.22	0.28
Ever smoked cigarettes								
No	4.8 (333)	–	–		0.3 (23)	–	–	
Yes	47.9 (457)	22.75	18.64 to 27.76	<0.01	10.0 (92)	66.30	39.52 to 111.23	<0.01
Frequency of current tobacco use								
I do not smoke	8.3 (637)	–	–	–	0.7 (54)	–	–	–
Less than once a week	51.3 (81)	14.78	10.13 to 21.55	<0.01	10.0 (14)	30.14	16.70 to 54.37	<0.01
At least once a week (but not every day)	52.0 (47)	22.98	13.51 to 39.10	<0.01	21.4 (21)	121.15	57.56 to 254.97	<0.01
Every day	58.3 (118)	32.00	23.45 to 43.83	<0.01	19.0 (36)	115.38	70.09 to 189.91	<0.01
Ever used cannabis								
No	7.8 (539)	–	–		0.7 (47)	–	–	
Yes	52.0 (290)	20.01	16.26 to 24.64	<0.01	12.6 (67)	53.03	34.87 to 80.65	<0.01

HBSC, Health Behaviour in School-aged Children; RRR, relative risk ratio.

## Discussion

Experimentation with e-cigarette use among young people in Wales is comparable in prevalence to experimentation with tobacco, with both rising steadily with age. By comparison to young people who had smoked tobacco, a lower percentage of never-smokers reported e-cigarette use. However, as overall, the group of never smokers was larger, in absolute terms it included almost as many young people who had used an e-cigarette a few times (though fewer regular users) as did the smaller pool of ever smokers. Hence, approximately half of young people who reported that they had used e-cigarettes also reported that they had never used tobacco. Indeed, among younger people under 14 the majority who had ever used an e-cigarette report that they have never smoked tobacco. It is possible that the higher prevalence of experimentation with e-cigarettes compared with tobacco use is because e-cigarettes are easier for young people to obtain given the lack of legal age restrictions on their sale; a situation which is currently being reviewed in some countries.^33^ However, it is important to note the low prevalence of *regular e-cigarette use*, which suggests that e-cigarettes are unlikely to make a major direct contribution to adolescent nicotine addiction at present.

Nevertheless, while much e-cigarette use appears to occur among young people who have never smoked tobacco, strong dose–response associations were observed between smoking and e-cigarette use, with secondary school students who currently smoke tobacco far more likely to use, or to have used, e-cigarettes. Associations of e-cigarette use with ever smoking could indicate that some young people have attempted to use e-cigarettes as a means of quitting smoking. While it appears that e-cigarettes can sometimes be used to help adult smokers stop smoking,^4^ ^5^ ^34^ to date their effectiveness as smoking cessation devices for non-adult populations has not been researched. However, if young people were effectively using e-cigarettes to support cessation one would expect to see a stronger association between ever smoking and e-cigarette use than between current smoking and e-cigarette use. Indeed, from the aforementioned harm reduction perspective,^6^ the ideal scenario would be for e-cigarette use to occur only among individuals who have previously smoked tobacco (ie, all young people who had ever used an e-cigarette would be ever smokers), and for e-cigarette use to replace tobacco smoking, rather than co-occurring (ie, no young people who had ever used an e-cigarette would also be current smokers of tobacco). The finding that current smoking was as strongly associated with e-cigarette use as was ever smoking indicates that young people are not adopting e-cigarettes as an effective means of quitting tobacco. However, it is important to note that the survey did not measure daily use of e-cigarettes, and that experimental use of e-cigarettes is likely to contribute little either to nicotine addiction or to smoking cessation efforts.

There are a number of possible explanations for the substantial overlap between e-cigarette use and current smoking. First, e-cigarette use and smoking may be driven by similar determinants and hence co-occur without being causally related to one another. Second, e-cigarettes might have been used by adolescent smokers with the intention of quitting smoking, but unsuccessfully. Third, the use of an e-cigarette may have preceded the establishment of current smoking behaviour, potentially acting as a ‘gateway’ into smoking.^7^ ^9^ The WHO have recognised that there is currently limited evidence regarding whether e-cigarettes may or may not act as a gateway into smoking tobacco.^22^ While two recent cross-sectional studies demonstrate a significant association between e-cigarette use and intention to smoke tobacco,^7^ ^9^ longitudinal studies are required to unpick the temporal relationships of e-cigarette and tobacco use.

There was limited evidence of patterning in adolescent e-cigarette use by sociodemographic factors (with the exception of marginally greater use among boys in the primary school sample). This is in very stark contrast to consistent evidence of a strong sociodemographic patterning in tobacco use among secondary-schools students according factors such as family affluence and ethnicity.^35^ ^36^ Public health professionals and policymakers are increasingly concerned that e-cigarettes will act as a new gateway into nicotine addiction and tobacco use for some young people if e-cigarettes are taken up widely at a population level. These data do suggest e-cigarette use could potentially spread throughout the youth population and become ‘normalised’, irrespective of socioeconomic status, ethnicity and gender, which was a feature of cannabis and ‘club drug’ use during the 1990s.^37–39^

This study benefits from two large, nationally-representative samples and, through combining these two data sets, extends analysis to a broad age range of young people, allowing analysis of e-cigarette use by age. However, a number of limitations should be considered. Measures of self-reported e-cigarette use are not validated, and hence the extent to which they capture true prevalence is unclear. Indeed, the fact that estimates of e-cigarette use were higher in the primary school survey than for younger people in the secondary school survey is likely due to differences in questionnaire wording. Within the HBSC, the item included response options of ‘never’, ‘a few times’ (defined as 1–5) or ‘regularly’. Young people who had used an e-cigarette once, but operated with the more common definition of a few (as a small number, but more than one), may have been more likely to select ‘never’, leading to underestimation of prevalence of ever use. A lack of unified definition of what constitutes ‘use’ of an e-cigarette is also a challenge for survey research, while measures should aim to also capture very regular (ie, daily) use. We would like to echo calls for improved survey measures for capturing e-cigarette use,^40^ including for research with young people. There were also some minor differences in response rates across the two surveys and they were not conducted at precisely the same time, although all data were collected across both studies within a 6-month period from November 2013 to April 2014. Finally, these cross-sectional data do not allow us to firmly identify whether increasing e-cigarette use by age reflects a development trajectory, or whether these reflect increases that have occurred in prevalence across all age groups as e-cigarettes have become more widespread. Longitudinal data are urgently needed to understand the temporal sequence of links between e-cigarette use, tobacco and cannabis use. It also worth noting that the context and nature of e-cigarette use among teenagers surveyed may be quite different to their use among the next generation who are the first to grow up with e-cigarettes. To understand this context and youth e-cigarette use in more depth mixed methods research will also be required.

## Conclusion

In Wales, 6% of 10–11 year olds and 12% of 11–16 year olds have used an e-cigarette at least once. Many experimental e-cigarette users have never smoked a cigarette, although most regular e-cigarette users had also smoked tobacco. The prevalence of experimental e-cigarette use, combined with few distinctions according gender or family background could allow e-cigarettes to become normalised relatively quickly with the youth population. However, at present, there is a very low prevalence of *regular use*, which suggests that e-cigarettes are unlikely to be making a significant direct contribution to adolescent nicotine addiction. Future research is needed to understand the motivations behind young people's experimentation with e-cigarette use and to understand the temporal relationships between use of e-cigarettes and tobacco.
